# Optimizing Horticulture Luminescent Solar Concentrators via Enhanced Diffuse Emission Enabled by Micro-Cone Arrays

**DOI:** 10.1021/acsami.4c01707

**Published:** 2024-05-15

**Authors:** Zhijie Xu, Martyna Michalska, Ioannis Papakonstantinou

**Affiliations:** † Photonic Innovations Lab, Department of Electronic and Electrical Engineering, University College London, London WC1E 7JE, U.K.; ‡ Manufacturing Futures Lab, Department of Mechanical Engineering, University College London, Queen Elizabeth Olympic Park, London E20 3BS, U.K.

**Keywords:** luminescent solar concentrators, horticulture, outcoupling efficiency, microcones, spectral conversion, bidirectional transmittance distribution function

## Abstract

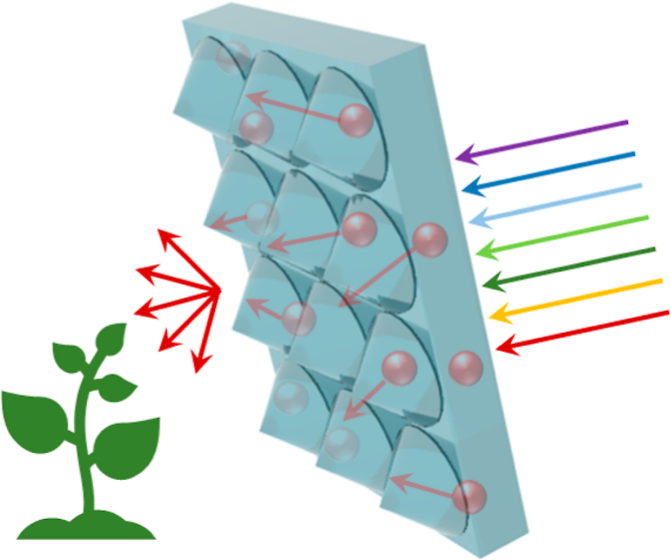

Optimizing the photon spectrum for photosynthesis concurrently with improving crop yields presents an efficient and sustainable pathway to alleviate global food shortages. Luminescent solar concentrators (LSCs), consisting of transparent host matrices doped with fluorophores, show excellent promise to achieve the desired spectral tailoring. However, conventional LSCs are predominantly engineered for photon concentration, which results in a limited outcoupling efficiency of converted photons. Here, we introduce a scheme to implement LSCs into horticulture (HLSC) by enhancing light extraction. The symmetry of the device is disrupted by incorporating microcone arrays on the bottom surface to mitigate total internal reflection. Both Monte Carlo ray tracing simulations and experimental results have verified that the greatest enhancements in converted light extraction, relative to planar LSCs, are achieved using microcone arrays (base width 50 μm, aspect ratio 1.2) with extruded and protruded profiles (85.15 and 66.55% improvement, respectively). Angularly resolved transmission measurements show that the HLSC device exhibits a broad angular radiation distribution. This characteristic indicates that the HLSC device emits diffuse light, which is conducive to optimal plant growth.

## Introduction

Light is recognized as one of the paramount factors influencing plant growth.^1−3^ Broadly, light’s influence can be categorized into three key aspects: (1) light quantity, (2) light photoperiod, and (3) light quality.^4^ Light quantity refers to the intensity of light received by plants within the photosynthetically active radiation (PAR) spectrum, which spans from 400 to 700 nm and encompasses the wavelengths relevant to horticultural practices. Photoperiod is the amount of time plants receive light during a 24 h period. Finally, light quality refers to the spectral distribution.^5−7^ In brief, chlorophyll *a* and *b*, molecular pigments integral to photosynthesis of plants, exhibit strong absorption of blue and red wavelengths.^8^ Conversely, ultraviolet and green can negatively impact crops by diminishing photosynthesis, decreasing shoot length, and reducing leaf absorption.^9^ In other words, some parts of the PAR spectrum and particularly red are more beneficial for plant growth.

**Figure 1:**
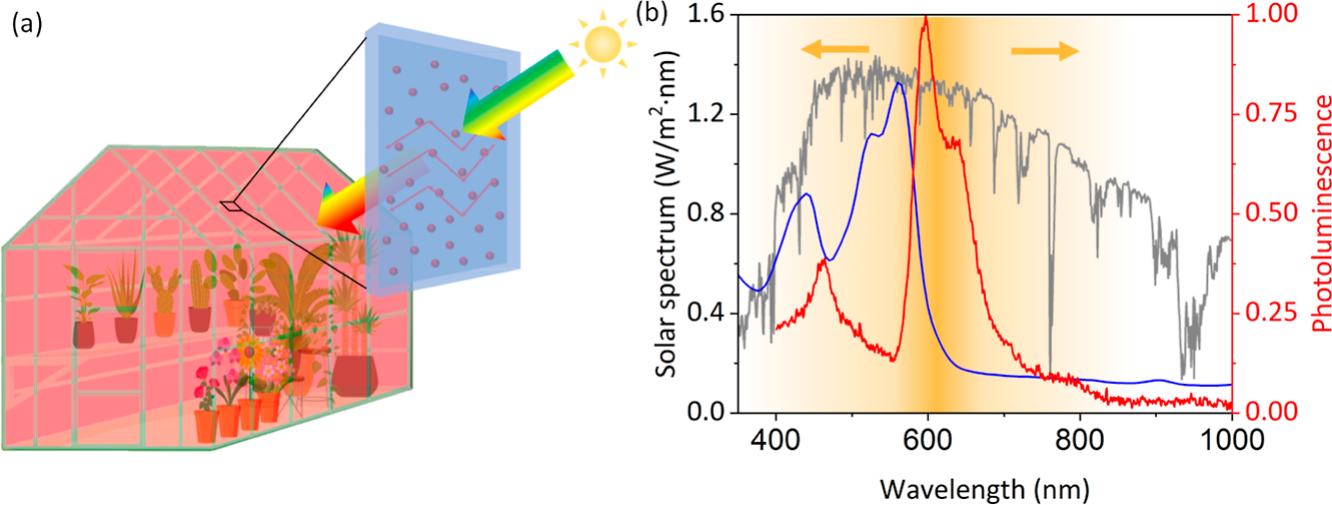
(a) Illustration of a greenhouse covered by HLSC. (b) Normalized absorption (blue line) and photoluminescence (red line) spectra of Lumogen Red in PDMS HLSC (dye concentration 1 × 10^–4^ M). Photoluminescence was measured upon excitation at 350 nm. The solar spectrum is represented by the gray line, while the benefit for photosynthesis is indicated by the shaded gold region, diminishing with variations in wavelength (the golden arrows depict the trend of decay).

To accelerate plant growth in greenhouses, artificial lighting, such as from LEDs,^10^ has extensively been used. In principle though, natural light could also be manipulated to provide ideal lighting conditions for enhanced growth. Interestingly, the solar spectrum peaks in the green, whereas, as mentioned, the most useful wavelength in the PAR zone for plant growth is the red–see Figure 1b. An elegant solution, to circumvent the misalignment between the two primary spectra, would be to shift the peak energy of AM1.5 from the green to the red. LSCs are widely employed spectral-shifting devices that can achieve this aim due to the Stokes shift exhibited by the luminescent materials (or fluorophores) they contain.^11,12^ As a result, several studies have recently appeared on the application of LSCs in horticulture.^7,13,14^ LSCs not only facilitate spectral conversion but also confer the advantage of providing diffuse light for plant growth, given that fluorophores emit photons isotropically. Numerous prior investigations have substantiated the preference for diffuse light in cultivation^15^ as unidirectional light is limited to certain portions of the plants, typically the leaves. Nonetheless, not only leaves but also stems and roots require solar radiation for optimal growth, a provision that is effectively met by diffuse light.

In a standard LSC device, fluorophores are hosted by a transparent dielectric material, such as polymer or glass.^11,12,16−23^ As a result of the high refractive index contrast between the LSC and its surrounding (typically air), most converted light is concentrated within the device via total internal reflection (TIR), and it is guided toward its edges.^24,25^ This is undesirable for our purposes, however, since the objective here is to maximize the density of photons escaping and reaching the plants. In summary, apart from spectral conversion, light extraction is an equally critical component, but this is not effectively met by most HLSC technologies today.

Given the recent strides in fluorophore development over the past few decades, identifying appropriate green-to-red fluorophores is no longer a formidable challenge.^26^ As a result, the primary gap that remains in achieving highly efficient HLSC lies in the implementation of novel light extraction techniques to enhance light quantity. In recent times, a multitude of techniques aimed at enhancing outcoupling efficiency have emerged within optical displays and OLED research.^13,27−32^ The fundamental principle underlying these techniques revolves around the expansion of escape cones and/or the disruption of TIR cones.^33^ The most straightforward approach to addressing this challenge involves reducing the refractive index of the host matrix, hence shrinking the TIR cone.^32,34^ Another approach involves the use of nanostructures, such as gratings, metasurfaces, and photonic crystals, which overall help diffract waveguide modes out of the device.^29,35,36^ A third option is the incorporation of micrometer relief structures on the surface of the LSC, which can enhance photon randomization, thereby creating more opportunities for light to enter the escape cone.^37−39^ Among these options, microstructures are probably the most compatible solution with HLSC due to their potential scalability using current manufacturing methods, such as roll-to-roll hot-embossing.^40^ Recently, a microlens array was applied to the top surface of an LSC device to extract about 30% of internally generated light from the bottom surface.^7^ Alternatively, microcone arrays, widely used in numerous fields, from light outcoupling in optical backlights to light incoupling in solar cells can be used.^41−43^ Inspired by these studies, microcone arrays are proposed here for HLSC purposes.

We employed Lumogen Red, an affordable and readily available organic dye for spectral conversion. It should be noted though that our light extraction designs are widely applicable and not bound to any specific fluorophore, rendering them a universal solution for HLSC research. Supported by Monte Carlo ray tracing calculations, microcone arrays were first designed and optimized for effective light extraction. Several masters based on optimal designs were subsequently fabricated by 2-photon polymerization (2PP). Exact copies of the master surface relief structures (from now on called microcone extrusions) were created by a double inversion process, while a single inversion step created a negative copy (termed microcone protrusions). Even though protrusions exhibited a slightly lower outcoupling efficiency compared to extrusions, this design can potentially demonstrate superior robustness.^44^ Microcone arrays for proof-of-concept (6 × 6 mm^2^) were imprinted onto the bottom surface of PDMS-LSC (thickness of the sample is 3 mm), culminating in the realization of HLSC. The selection of PDMS as the host material, similar to the choice of fluorescent materials, is primarily attributed to its availability and cost-effectiveness, rather than implying that PDMS is the exclusive material suitable for HLSC research. The enhancements in outcoupling efficiency for the converted red-light amounted to 85.15 and 66.55% for the best microcone extrusion and protrusion, respectively. Remarkably, angular distribution measurements demonstrated that light was emitted into a broad range of angles, a beneficial characteristic for promoting plant growth. Hence, our study paves the way for the practical implementation of the HLSC technology.

## Results and Discussion

The schematic of HLSC realizing spectral conversion and improving photosynthesis is displayed in Figure 1a. Our proposed strategy involves employing fluorophores to capture green light and subsequently transform it into red light. Ideally, such a conversion should not compromise the photon counts of the solar radiation but rather optimize its spectral distribution. It is for this reason that fluorophores with high quantum efficiency are preferable. The absorption spectrum of Lumogen Red within HLSC was assessed using ultraviolet–visible spectroscopy (UV–vis, Shimadzu, UV-3600i Plus UV–vis–NIR spectrophotometer), revealing a peak around 560 nm, as shown in Figure 1b. Additionally, the emission spectrum was determined through time-correlated single photon count spectroscopy (Edinburgh Instruments, FLS1000 Photoluminescence Spectrometer), indicating a peak approximately at 595 nm. Alongside a Stokes shift of 35 nm from green to red light, the majority of re-emitted photons falls within the 600 to 700 nm range. This range aligns remarkably well with the most effective spectral range for photosynthesis, as depicted by a shaded gold region in Figure 1b. To quantitatively illustrate this spectral conversion, we compared the proportion of energy in the 600–700 nm wavelength range before and after conversion within the entire PAR spectrum. This proportion increases from 33.6% in the AM1.5 spectrum to 46.1% in the converted spectrum. Furthermore, within the entire emission spectrum of Lumogen Red, the power in PAR constitutes a substantial proportion, accounting for up to 85.9%, thereby further enhancing the spectral distribution of light received by plants.

**Figure 2:**
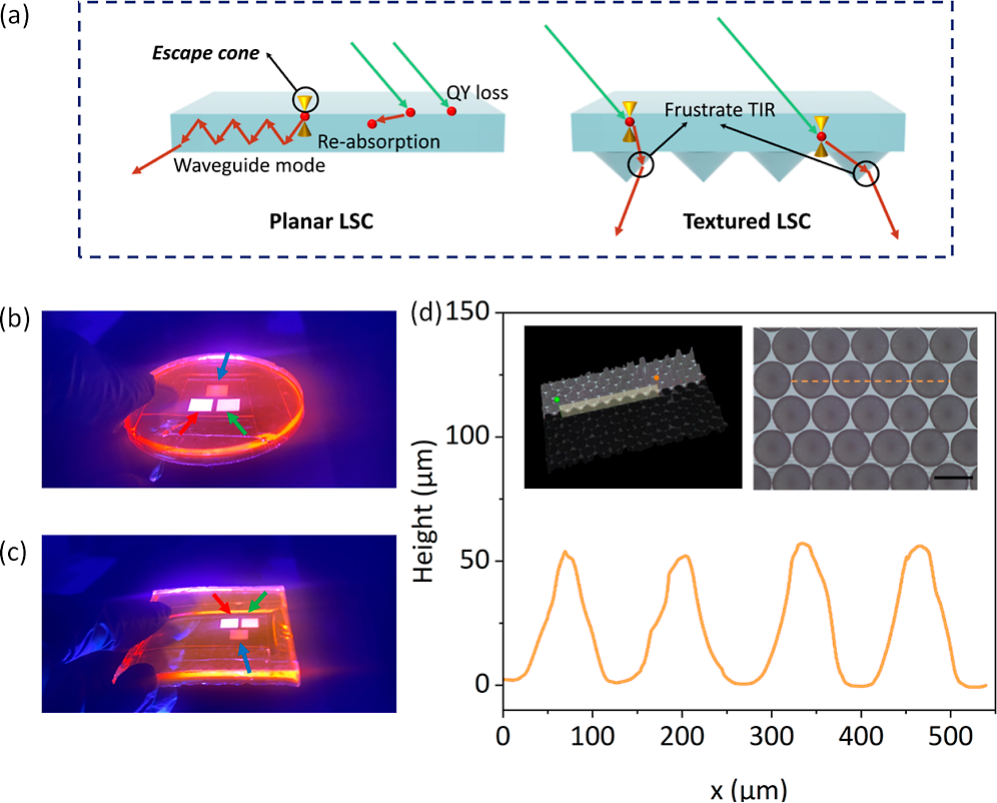
(a) Theoretical explanations of photon pathways in LSC and HLSC enhancing light extraction using microcone arrays. (b,c) Photographs illustrating the impact of (b) microcone extrusion and (c) protrusion on improving light extraction under ultraviolet illumination. (d) Microcone array morphology examination utilizing a 3D optical microscope. The left inset presents a 3D optical micrograph, while the right inset provides a top-view perspective. Scale bar: 100 μm.

While planar LSCs incorporating Lumogen Red can still attain efficient green-to-red conversion, their inherent ability to concentrate light constrains their applicability in horticulture. The possible photon fates in LSC devices are illustrated in Figure 2a.^23^ In this context, we undertook a re-evaluation of the importance of photon fates, which differ from those typically reported in LSC research. This is because in conventional LSCs, the waveguide mode represents the preferred photon pathway, whereas the extracted photons from the bottom surface are the preferred photon fate in HLSC. As depicted in Figure 2a, if a photon is emitted outside the escape cone, it is trapped due to TIR. The escape cone forms two pairs of symmetrical cones for emission angles smaller than the critical angle. Absorption serves an ambivalent role. On the one hand, high absorption effectively captures more light and transforms it into the desired range. On the other hand, it can result in undesirable high reabsorption rates.

Based on the preceding discussion, it becomes imperative to establish definitions for photon fates within the context of HLSC, distinct from those applicable to conventional LSC devices. To be more specific, in the HLSC context, the internal quantum efficiency (IQE) is defined as the percentage of photons that escape from the bottom surface to the total number of absorbed photons. Concurrently, top loss and edge loss refer to the proportion of photons that escape from the top and edge surfaces, respectively, in relation to the overall number of absorbed photons. The external quantum efficiency (EQE), on the other hand, characterizes the ratio of photons escaping from the bottom surface to the total number of incident photons. Likewise, external top loss and external edge loss signify the ratios of photons emanating from the top and edge surfaces relative to the total number of incident photons. Finally, unabsorbed loss designates the portion of photons traversing the device without undergoing absorption by fluorescent materials.

Building upon the aforementioned analysis of photon pathways in HLSC, the key challenge in achieving high-efficiency HLSC lies in mitigating the impact of the waveguide mode. Considering isotropic emission for the fluorophores, a substantial portion of photons—often exceeding 70%—tends to enter the waveguide mode for most common host materials, for which their refractive index is *n* ∼1.5.^45^ Therefore, incorporating a light extraction technique is necessary to mitigate light trapping.

Extruding and protruding microcone arrays, like the ones shown in Figure 2a, are validated as effective microstructures for extracting light from high-index host matrices. This approach has the capability to disrupt the device’s symmetry and promote the light outcoupling. This is because even if a photon is originally emitted within the TIR cone, the reflectance angle would gradually change upon encountering the microcone array, until it falls into the escape cone. More details of photon paths are depicted in Figure S1 in Supporting Information.

We employed Monte Carlo ray tracing to identify the optimal structural parameters. Subsequent analysis determined that an HLSC device featuring a height-to-radius ratio (H/R) of 1.2 and a fluorophore concentration of 1 × 10^–4^ M achieves the most favorable light extraction performance. The sample thickness is 3 mm, and the cone radius is fixed at 50 μm. The rationale for the selection of these parameters will be discussed in detail in subsequent sections. Simulated internal and external photon fates are depicted in Figure S2.

**Figure 3:**
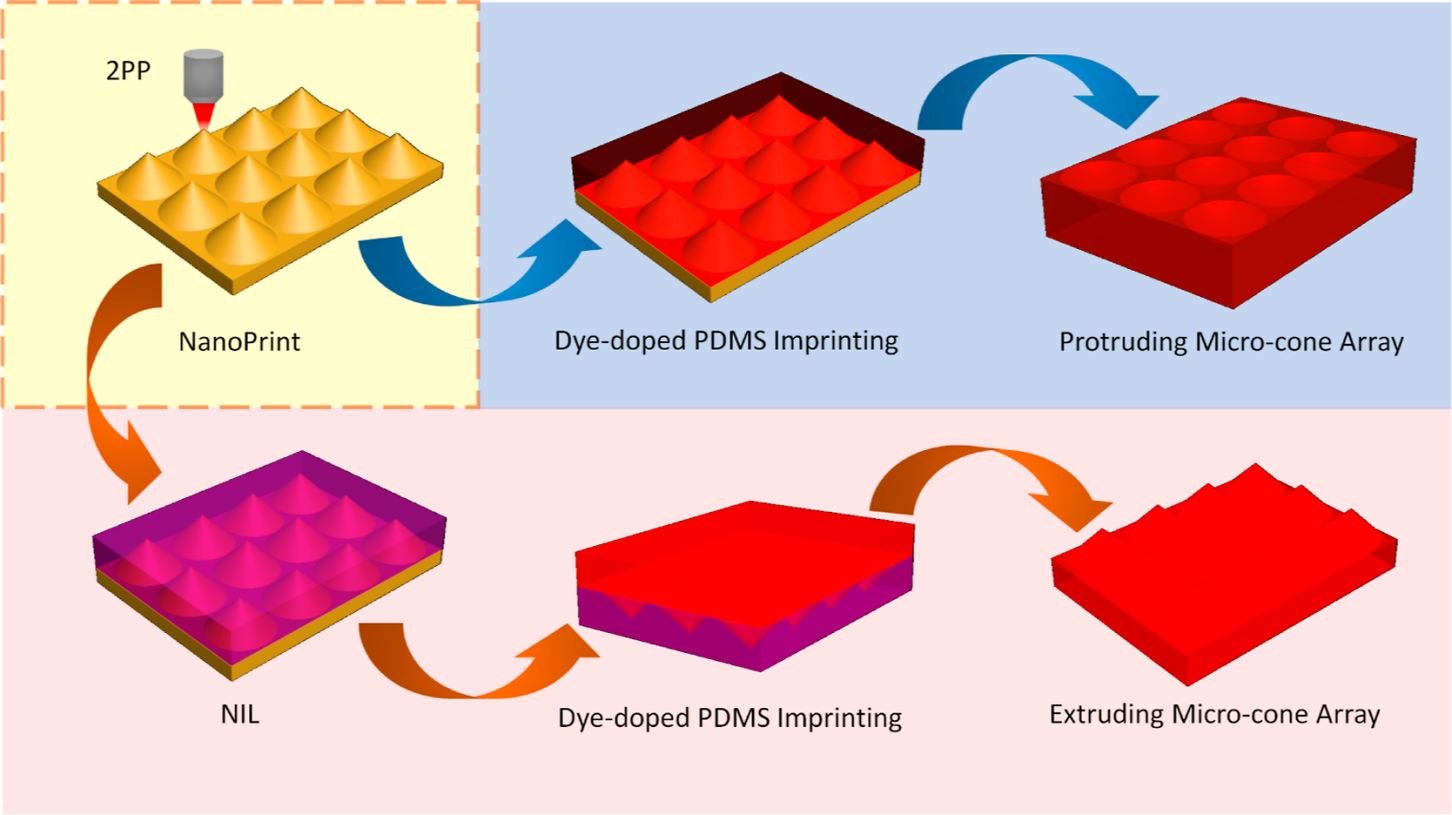
Illustrations depicting the fabrication process of HLCs with microcone extrusions and protrusions. The blue area and arrows indicate the fabrication process of protruding arrays, while the red area and arrows indicate the process of extruding arrays. The nanoprinted master for these two processes is marked within the yellow area surrounded by dashed line.

To fabricate HLSCs with microcone extrusions and protrusions, a multistep fabrication approach was employed. As illustrated in Figure 3, periodic microcone arrays were first 3D-printed onto a polished silicon (Si) wafer by 2PP (Photonic Professional GT, Nanoscribe). Following this step, the Si wafer with the microcone arrays served as a template to transfer the pattern into dye-doped PDMS via soft-lithography, ultimately producing protruding HLSC. Alternatively, nanoimprint lithography (NIL) was employed to first imprint protruding microcone arrays into an intermediate polymer stamp (IPS). Following a double inversion process using soft lithography in doped PDMS, an exact copy of the original extruding microcone arrays was created. Through the manipulation of the H/R ratio and fluorophore concentration, it becomes possible to tailor HLSCs to exhibit varying light extraction performances. For comparison, microcone arrays featuring three distinct H/R ratios (0.4, 1.2, and 2.0) and three different concentrations (1 × 10^–4^, 3 × 10^–5^, and 1 × 10^–5^ M) were fabricated.

The surface morphology of the microcone arrays was assessed using a 3D optical microscopy system (Keyence, VHX 5000). A cross-section of four microcones, featuring a radius of 50 μm and a height of 60 μm (yielding an H/R ratio of 1.2), is illustrated in Figure 2d. This outcome underscores the successful realization of our structure design through nanoprinting, NIL, and PDMS soft-lithography processes. The left inset within Figure 2d provides a 3D profile of the microcone array from a 45° viewing angle. Furthermore, the right inset presents a top view of the microcone array, offering a glimpse of the cone base profile. Additional characterization of the structures is provided in the Supporting Information.

Given that the fluorophores within HLSC are capable of absorbing incident photons from all directions, we utilize normally incident light to showcase the characteristics of HLSC. Additional Monte Carlo calculations have confirmed that the internal photon fates of HLSC under different incident angles remain nearly identical (see Figure S4). The outcoupling performance of the fabricated HLSCs was assessed by measuring the transmitted photon counts from the bottom surface. Visual representation of light extraction is provided in Figure 2b,c through photographs. The photographs clearly demonstrate that both microcone extrusions and protrusions exhibit significantly higher brightness compared to the surrounding planar LSC area. In the images, red arrows indicate the areas patterned by microcone arrays with an H/R ratio of 1.2, green arrows mark the areas with an H/R ratio of 2.0, and blue arrows designate the areas with an H/R ratio of 0.4. It is evident that there is a noticeable increase in brightness from the blue areas to the green areas and finally to the red areas, which is consistent with the quantitative results obtained in the subsequent analysis.

**Figure 4:**
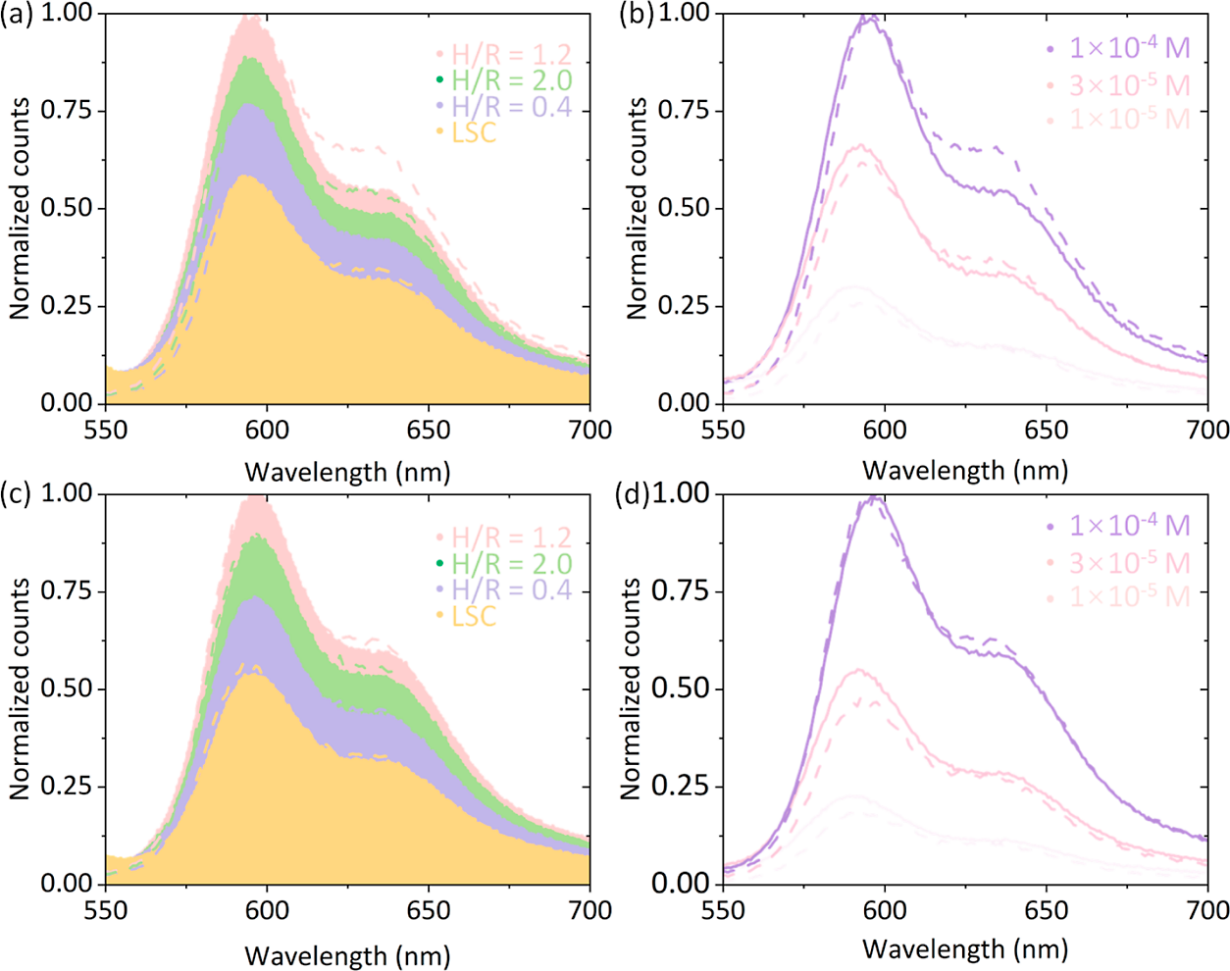
Normalized counts of photons emitted from bottom surface. (a) Comparison between protruded microcone arrays with different H/R ratios and planar LSC. (b) Comparison between different concentrations for protrusion. (c) Comparison between extruded microcone arrays with different H/R ratios and planar LSC. (d) Comparison between different concentrations for extrusion. Shades regions represent the outcoupling improvement. All solid lines correspond to the experimental data, while dashed lines represent simulations.

Photon counts were recorded using an integrating sphere (Labsphere, RTC-060-SF). To ensure fair comparison, microcone arrays with varying H/R values and a reference LSC were integrated into the same sample. All results have been normalized to the peak photon count. We integrated the photon counts between 550 and 700 nm for comparing outcoupling efficiency. The results, as illustrated in Figure 4a,c (solid lines), reveal substantial enhancements in the outcoupling efficiency for both extruding and protruding structures, reaching 85.15 and 66.55%, respectively, compared to planar LSCs. In detail, 43.09% (extrusion) and 41.91% (protrusion) of the converted red photons can escape from the bottom surface (see Figure S2), percentages that are significantly higher than those achieved with microlens arrays (approximately 30%).^7^ Despite the structural differences, the light loss channels of HLSC only involve the waveguiding mode. However, compared to traditional OLED light extraction techniques, microcone arrays not only maintain a high extraction efficiency of converted photons but also possess the advantage of easy large-area processing.

Notably, all measured outcomes exhibit good agreement with Monte Carlo ray tracing predictions, as indicated by the dashed lines in Figure 4a,c. Across all H/R values, microcone arrays consistently display superior outcoupling efficiency when compared to planar LSCs. Of particular significance, the most substantial enhancement is observed at an H/R ratio of 1.2. This enhancement can be attributed to the combined effect of higher absorption and IQE. As previously established,^33^ EQE is influenced by both IQE and absorbance according to the following equation
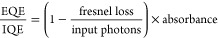
1

As can be seen from the eq 1, enhancing absorbance and IQE simultaneously hold the potential to enhance the EQE of the device. As outlined in Figure S2, the results from Monte Carlo ray tracing affirm that an H/R ratio of 1.2 leads to the optimal combination of absorption and IQE for microcone arrays. This is because absorption exhibits a maximum at H/R = 1.2, while IQE saturates for H/R = 1.2. The combination of the above two conditions results in a maximum for the EQE also at H/R = 1.2.

As shown in Figure S1, in the case of a high H/R ratio, even when the initial incident angle is greater than the critical angle, the microcone structure effectively reduces the incident angle through subsequent reflections. This ultimately results in the incident angle becoming smaller than the critical angle, allowing the photons to escape from the device. The results from Figure S2 indicate that a continuous increase in the H/R ratio leads to saturation in the improvement of IQE, reaching a critical threshold (H/R ∼1).

Remarkably, a higher concentration is observed to yield superior performance, despite its potential for inducing increased reabsorption, as indicated in Figure 4b,d. This finding diverges somewhat from earlier research on LSCs, potentially simplifying future HLSC design by reducing the need for meticulous concentration optimization efforts. Furthermore, due to the pronounced reabsorption effect, the emission peak exhibits a notable red-shifting. Furthermore, when the concentration is 1 × 10^–4^ M, the absorbance of HLSC reaches about 96% (excitation wavelength is 520 nm). As a result, increasing the concentration further may not be a more effective means of improving the performance of HLSC (see Figure S5). When the concentration is increased to 2 × 10^–4^ M, both internal and external photon fates remain unchanged. However, if the concentration is raised to 3 × 10^–4^ M or even higher, further increasing the concentration yields diminishing returns in terms of enhancing the device’s overall performance.

In order to better understand the effects of the dye concentration on reabsorption and photon fates, we conducted additional simulations to cover a wider range of concentration variations, as shown in Figure S5. QY loss serves as a robust indicator of reabsorption as the increase in QY loss mainly arises from an increase in reabsorption under otherwise unchanged conditions. From Figure S5, it can be observed that with the increase in the dye concentration, reabsorption, indicated by QY loss, significantly increases. Furthermore, as the dye concentration increases from around 2 × 10^–5^ to 1 × 10^–4^ M, the IQE and EQE reach a peak. This is because, with the increase in concentration, more photons are absorbed, concomitantly resulting in more photons being able to exit the device from the bottom. However, as the concentration further increases, the IQE and EQE begin to decline. This is due to the fact that although the increase in the concentration leads to higher absorption, a bigger fraction of reabsorbed photons is lost as heat due to nonunity QY. On the other hand, even photons that can be re-emitted have a high probability of being reabsorbed again. As re-emitted photons are isotropic, the probability of re-emitted photons being emitted toward the top surface or bottom surface is equal. Multiple reabsorptions inevitably hinder the movement of photons from the top region to the bottom region, making it difficult for them to exit from the bottom surface, thereby resulting in a decrease in IQE and EQE.

**Figure 5:**
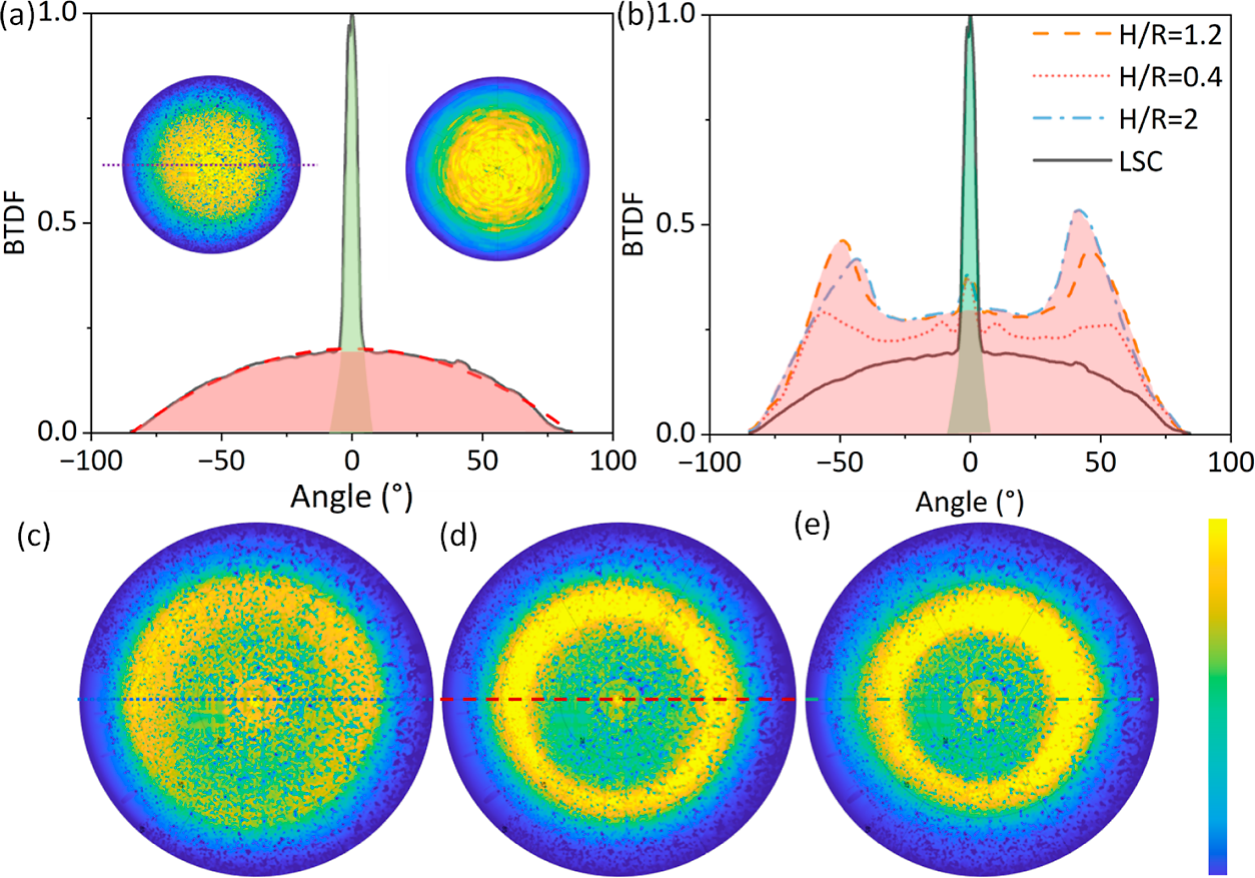
(a) BTDF of planar LSC. Solid black line is the measured result from planar LSC, and dash red line is its Lambertian fitting. The insets show the BTDF of experiment and simulation. (b) Cross sections of BTDF of different H/R ratios and planar LSC. Red areas in (a,b) represent emission, while the green areas depict the remaining excitation. (c–e) BTDF of different H/R ratios.

In a typical scenario, fluorophores emit light in an isotropic manner. Consequently, a planar LSC device would be akin to a Lambertian light source. To confirm this angular distribution, bidirectional transmittance distribution function (BTDF, IS-SA, Radiant Zemax) measurements were conducted upon excitation at 350 nm, as demonstrated in Figure 5a. The measured BTDF is presented in polar coordinates (left inset) and agrees very well with Monte Carlo simulations (right inset). Both results are normalized to the peak irradiance for direct comparison. The cross-section along the dashed line on the measured BTDF is also plotted (black line) in Figure 5a and compared with an ideal Lambertian profile (red dashed line. The result confirms that the light output from the LSC follows an almost perfect Lambertian profile), see the red area in Figure 5a. Note that the peak observed in the experimental cross-section arises from the part of the incident excitation that has not been absorbed by the HLSC (the green area in Figure 5a,b). Since incidence is at a normal angle, a delta function appears at 0°. The reduction in the 0° peaks in Figure 5b, corresponding to the incident excitation from LSC to textured HLSC, serves as confirmation of the enhanced absorption of green light.

The angular distribution of HLSC is also both measured and simulated. The comprehensive irradiance profiles are displayed in Figure 5c–e. Contrasting with the BTDF profile of the planar LSC, the most striking difference is the emergence of secondary rings at angles of 57, 49, and 42° for H/R 0.4, 1.2, and 2.0, respectively. This can be attributed to the geometry of the microcone arrays, which exhibit a preference for extracting light at angles nearly perpendicular to their surface. As H/R changes from 0.4 to 2.0, the cone angle decreases from 136 to 53°, resulting in a shift to the position of the secondary rings. The detailed ray tracing results in Figure S1 further explain the process. Furthermore, the overall irradiances stemming from the microcone arrays surpass those of the planar LSC across all emission angles. This outcome provides further confirmation of the enhancement in outcoupling efficiency through angular distribution. These distributions corroborate the capacity of HLSC to offer diffuse light conducive to plant growth. Additionally, the QY of the HLSC remained almost unchanged throughout the UV resistance test, as depicted in Figure S11. This observation serves as further evidence of the exceptional light stability exhibited by the sample.

As previously mentioned, aside from light quality, crop yield is also influenced by light quantity. This parameter is intricately tied to the direction of incident light. However, this is constantly changing throughout the day. For this reason, it is important to evaluate the performance of HLSC under different angles of incidence. As a realistic scenario, we used London to illustrate the correlation between incident angle and insolation—a parameter frequently employed to characterize the radiation reaching the Earth’s surface. As depicted in Figure S7, if the incident angle falls above 45°, there is a marked decline in insolation. Consequently, our attention is directed toward incident angles below 50°, a range sufficient for effectively harnessing solar energy.

**Figure 6:**
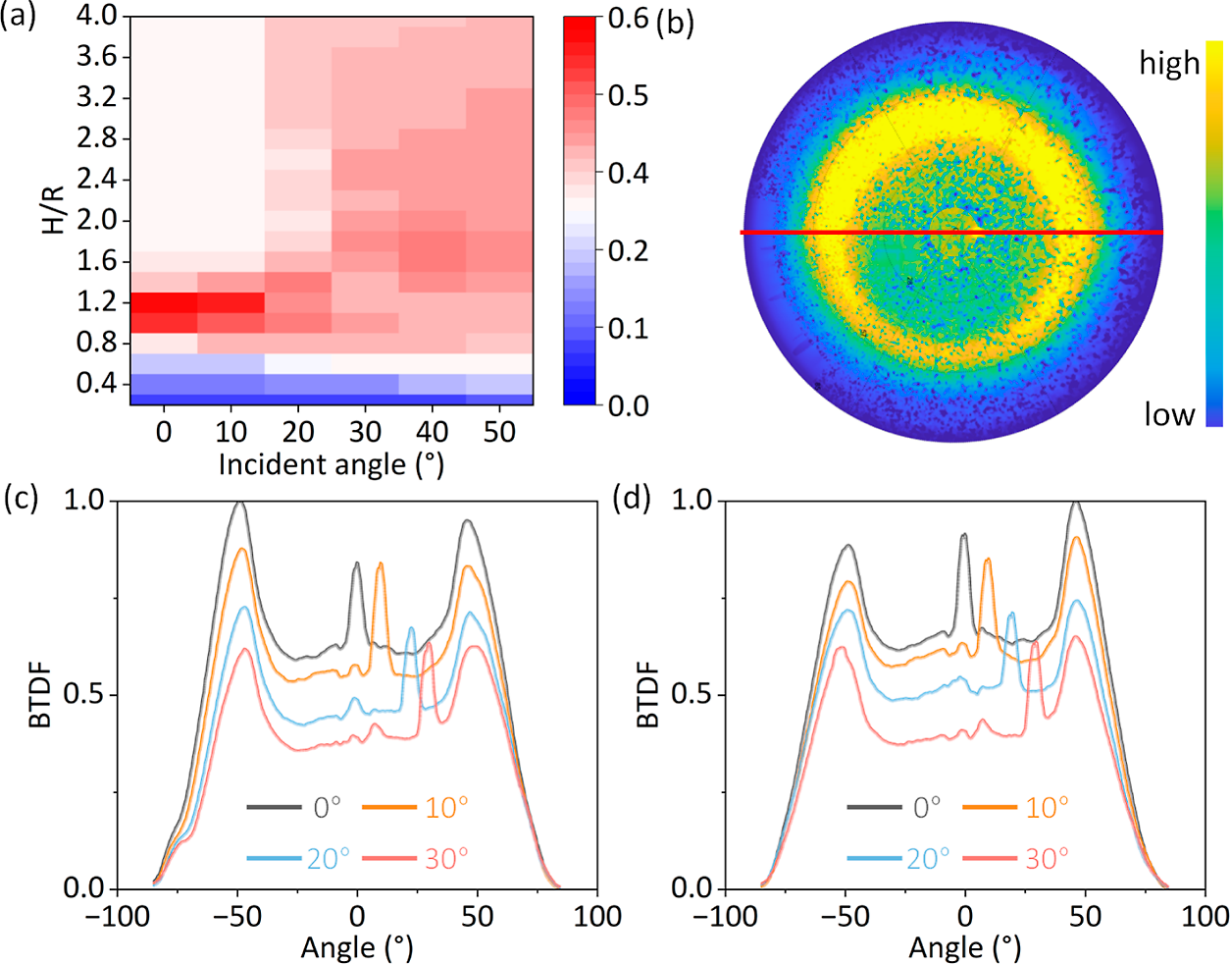
(a) EQE enhancement for different H/R under distinct incident angles. (b) BTDF in polar coordinates for microcone extrusions under an incident angle of 10°. (c,d) Cross sections of BTDF under different incident angles for (c) extruding and (d) protruding microcone arrays.

The enhancement of EQE, in comparison to a planar LSC is presented in Figure 6a. Results reveal that HLSCs attains the highest EOE improvement, particularly when the H/R ratio is 1.2, across the majority of incident angles ranging from 0 to 50°. This finding reinforces the selection of this parameter as the optimal choice for incident angles. Furthermore, BTDF distributions are also calculated for varying incident angles, in Figure 6. The profiles of irradiance resemble those observed under normal incident conditions (see Figure S8), thereby continuing to provide diffuse light conducive to plant growth.

**Figure 7:**
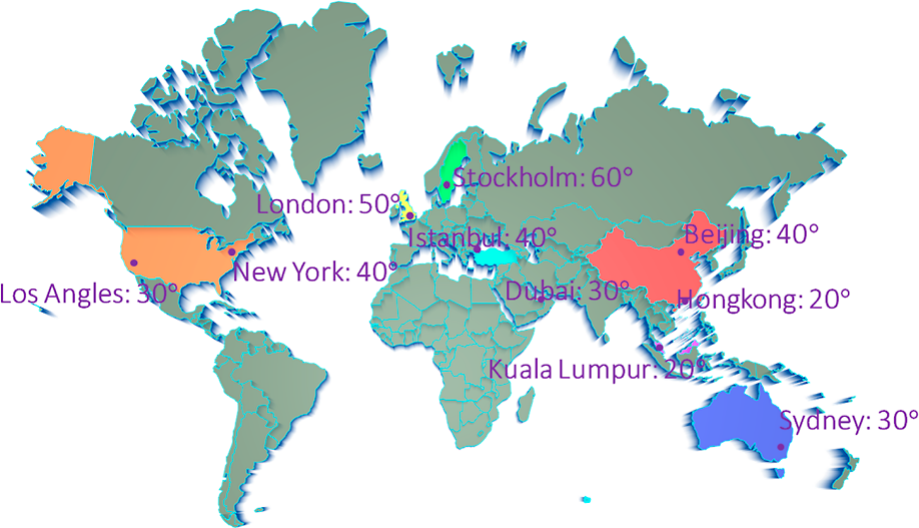
Recommended roof slopes of HLSC in different areas of world to achieve best photosynthesis efficiency.

To ensure that HLSC predominantly captures light from directions below 45°, we offer our recommendations for greenhouse roof slopes in various regions worldwide, as illustrated in Figure 7. As indicated by eqs S1–S8, insolation levels in various geographical regions are predominantly influenced by their respective latitudes. Consequently, the optimal roof slope varies in response to these latitude differences. This relationship is depicted in Figure 7, which illustrates that cities sharing a similar latitude tend to exhibit similar recommended roof slopes. For instance, regions located at lower latitudes, such as Kuala Lumpur, typically have relatively shallow roof slopes. In contrast, cities situated at higher latitudes, such as Stockholm, are expected to require steeper roof slopes to maximize solar exposure.

## Conclusions

In summary, through the utilization of microcone array patterned HLSC, we have showcased a significant improvement in the outcoupling efficiency of converted red light. The incorporation of multistep manufacturing techniques involving nanoprint and NIL has been detailed for the fabrication of PDMS-based HLSC devices. The collective evidence from both experimental and simulation results attests to the enhanced light extraction performance when compared to planar LSCs. Additionally, the measured angular distributions of HLSC devices underscore their capability to provide diffuse light, contributing to the optimal growth of plants.

## Experimental Section

### Nanoprint and NIL

Microcone arrays with various H/R ratios are fabricated on a meticulously polished silicon wafer (25 × 25 × 0.725 mm^3^). The wafer undergoes a thorough cleaning process using IPA and acetone solvents. Subsequently, it is placed within a vacuum chamber for a plasma treatment lasting 60 s. The formation of microcone arrays on the silicon wafer is achieved through 2PP using a ×10 objective lens and a highly viscous liquid negative-tone resin known as IP-Q. Both the silicon wafer and IP-Q resin were procured from NanoScribe. Recognizing that 2PP is a considerably time-intensive procedure, modifications were made to the microcone arrays, transforming them into scaffolding structures. This adjustment allows for the retention of the microcone profiles for subsequent imprinting while significantly reducing the polymerization time.

IPS is employed to replicate the microcone arrays obtained via nanoprint through a NIL process. The sample is introduced into the nanoimprint facility (EITRE3, Obducat), where the imprinting process is conducted. The temperature is set to 170 °C, and a pressure of 20 bar is applied for 60 s to execute the imprinting. Subsequently, the temperature is reduced to 100 °C for 20 s. Finally, the temperature is further lowered to 70 °C, and the pressure is released to atmospheric levels. The entire NIL process takes approximately 10 min, significantly shorter than the nanoprint process, which typically requires around 6 h to cover a 6 × 6 mm^2^ area. Additionally, the use of IPS offers advantages in terms of durability and cost-effectiveness, making it well-suited for large-scale manufacturing.

### HLSC Fabrication

Lumogen Red (Sun Chemical Limited) is dissolved in ethyl acetate, which is compatible with PDMS. The dye solution is then added to the elastomer at the desired concentration and mixed using a magnetic stirrer. Subsequently, the mixture is placed in an ultrasonic bath at a temperature of 6 °C for 1 h. Following this, the curing agent (Sylgard 184, Dow Corning) is introduced into the mixture in a weight ratio of 1:10 compared to the elastomer. After degassing, the mixture is poured into a glass mold, the bottom surface of which is covered with either the nanoprint or NIL sample. The mold is then placed on a hot plate set at a temperature of 60 °C for 2 h to produce the final HLSC.

## Supporting Information Available

Ray path in the microcone structure; internal and external photon fates; microcone array morphologies; internal photon fates under different incident angles; concentrations of HLSC; radiation angle and insolation calculation; insolation variation; Monte Carlo ray tracing; and simulated HLSC BTDF (PDF)

## References

[ref1] Kelly N.; Choe D.; Meng Q.; Runkle E. S. Promotion of Lettuce Growth under an Increasing Daily Light Integral Depends on the Combination of the Photosynthetic Photon Flux Density and Photoperiod. Sci. Hortic. 2020, 272, 109565 10.1016/j.scienta.2020.109565.

[ref2] Xu W.; Lu N.; Kikuchi M.; Takagaki M. Continuous Lighting and High Daily Light Integral. Plants 2021, 10, 1203 10.3390/plants10061203. 34204820 PMC8231634

[ref3] Faust J. E.; Holcombe V.; Rajapakse N. C.; Layne D. R. The Effect of Daily Light Integral on Bedding Plant Growth and Flowering. HortScience 2005, 40 (3), 645–649. 10.21273/HORTSCI.40.3.645.

[ref4] Paradiso R.; Proietti S. Light-Quality Manipulation to Control Plant Growth and Photomorphogenesis in Greenhouse Horticulture: The State of the Art and the Opportunities of Modern LED Systems. J. Plant Growth Regul. 2022, 41, 742–780. 10.1007/s00344-021-10337-y.

[ref5] Wondraczek L.; Batentschuk M.; Schmidt M. A.; Borchardt R.; Scheiner S.; Seemann B.; Schweizer P.; Brabec C. J. Solar Spectral Conversion for Improving the Photosynthetic Activity in Algae Reactors. Nat. Commun. 2013, 4, 2047 10.1038/ncomms3047. 23797513

[ref6] Ooms M. D.; Dinh C. T.; Sargent E. H.; Sinton D. Photon Management for Augmented Photosynthesis. Nat. Commun. 2016, 7, 12699 10.1038/ncomms12699. 27581187 PMC5025804

[ref7] Shen L.; Lou R.; Park Y.; Guo Y.; Stallknecht E. J.; Xiao Y.; Rieder D.; Yang R.; Runkle E. S.; Yin X. Increasing Greenhouse Production by Spectral-Shifting and Unidirectional Light-Extracting Photonics. Nat. Food 2021, 2 (6), 434–441. 10.1038/s43016-021-00307-8. 37118233

[ref8] Park Y.; Runkle E. S. Spectral-Conversion Film Potential for Greenhouses: Utility of Green-to-Red Photons Conversion and Far-Red Filtration for Plant Growth. PLoS One 2023, 18, e0281996 10.1371/journal.pone.0281996. 36821557 PMC9949677

[ref9] Meyer P.; Van de Poel B.; De Coninck B. UV-B Light and Its Application Potential to Reduce Disease and Pest Incidence in Crops. Hortic. Res. 2021, 8, 194 10.1038/s41438-021-00629-5. 34465753 PMC8408258

[ref10] Bantis F.; Smirnakou S.; Ouzounis T.; Koukounaras A.; Ntagkas N.; Radoglou K. Current Status and Recent Achievements in the Field of Horticulture with the Use of Light-Emitting Diodes (LEDs). Sci. Hortic. 2018, 235, 437–451. 10.1016/j.scienta.2018.02.058.

[ref11] Ferreira R. A. S.; Correia S. F. H.; Monguzzi A.; Liu X.; Meinardi F. Spectral Converters for Photovoltaics - What’s Ahead. Mater. Today 2020, 33, 105–121. 10.1016/j.mattod.2019.10.002.

[ref12] Papakonstantinou I.; Portnoi M.; Debijie M. G. The Hidden Potential of Luminescent Solar Concentrators. Adv. Energy Mater. 2021, 11, 2002883 10.1002/aenm.202002883.

[ref13] Hammam M.; El-Mansy M. K.; El-Bashir S. M.; El-Shaarawy M. G. Performance Evaluation of Thin-Film Solar Concentrators for Greenhouse Applications. Desalination 2007, 209, 244–250. 10.1016/j.desal.2007.04.034.

[ref14] Keil J.; Liu Y.; Kortshagen U.; Ferry V. E. Bilayer Luminescent Solar Concentrators with Enhanced Absorption and Efficiency for Agrivoltaic Applications. ACS Appl. Energy Mater. 2021, 4 (12), 14102–14110. 10.1021/acsaem.1c02860.

[ref15] Li T.; Yang Q. Advantages of Diffuse Light for Horticultural Production and Perspectives for Further Research. Front. Plant Sci. 2015, 6, 704 10.3389/fpls.2015.00704. 26388890 PMC4559655

[ref16] Portnoi M.; Sol C.; Tummeltshammer C.; Papakonstantinou I. Impact of Curvature on the Optimal Configuration of Flexible Luminescent Solar Concentrators. Opt. Lett. 2017, 42 (14), 2695 10.1364/OL.42.002695. 28708146

[ref17] Tummeltshammer C.; Taylor A.; Kenyon A. J.; Papakonstantinou I. Flexible and Fluorophore-Doped Luminescent Solar Concentrators Based on Polydimethylsiloxane. Opt. Lett. 2016, 41 (4), 713 10.1364/OL.41.000713. 26872170

[ref18] Tummeltshammer C.; Taylor A.; Kenyon A. J.; Papakonstantinou I. Homeotropic Alignment and Förster Resonance Energy Transfer: The Way to a Brighter Luminescent Solar Concentrator. J. Appl. Phys. 2014, 116 (17), 173103 10.1063/1.4900986.

[ref19] Tummeltshammer C.; Brown M. S.; Taylor A.; Kenyon A. J.; Papakonstantinou I. Efficiency and Loss Mechanisms of Plasmonic Luminescent Solar Concentrators. Opt. Express 2013, 21 (S5), A735 10.1364/OE.21.00A735. 24104570

[ref20] Portnoi M.; Haigh P. A.; Macdonald T. J.; Ambroz F.; Parkin I. P.; Darwazeh I.; Papakonstantinou I. Bandwidth Limits of Luminescent Solar Concentrators as Detectors in Free-Space Optical Communication Systems. Light: Sci. Appl. 2021, 10 (1), 3 10.1038/s41377-020-00444-y. 33386386 PMC7775919

[ref21] Portnoi M.; Macdonald T. J.; Sol C.; Robbins T. S.; Li T.; Schläfer J.; Guldin S.; Parkin I. P.; Papakonstantinou I. All-Silicone-Based Distributed Bragg Reflectors for Efficient Flexible Luminescent Solar Concentrators. Nano Energy 2020, 70, 104507 10.1016/j.nanoen.2020.104507.

[ref22] Tummeltshammer C.; Portnoi M.; Mitchell S. A.; Lee A. T.; Kenyon A. J.; Tabor A. B.; Papakonstantinou I. On the Ability of Förster Resonance Energy Transfer to Enhance Luminescent Solar Concentrator Efficiency. Nano Energy 2017, 32, 263–270. 10.1016/j.nanoen.2016.11.058.

[ref23] Tummeltshammer C.; Taylor A.; Kenyon A. J.; Papakonstantinou I. Losses in Luminescent Solar Concentrators Unveiled. Sol. Energy Mater. Sol. Cells 2016, 144, 40–47. 10.1016/j.solmat.2015.08.008.

[ref24] Goetzberger A.; Greube W. Solar Energy Conversion with Fluorescent Collectors. Appl. Phys. 1977, 14 (2), 123–139. 10.1007/BF00883080.

[ref25] Weber W. H.; Lambe J. Luminescent Greenhouse Collector for Solar Radiation. Appl. Opt. 1976, 15 (10), 2299 10.1364/AO.15.002299. 20165383

[ref26] Desmet L.; Ras A. J. M.; de Boer D. K. G.; Debije M. G. Monocrystalline Silicon Photovoltaic Luminescent Solar Concentrator with 42% Power Conversion Efficiency. Opt. Lett. 2012, 37 (15), 3087 10.1364/OL.37.003087. 22859094

[ref27] Mao P.; Liu C.; Li X.; Liu M.; Chen Q.; Han M.; Maier S. A.; Sargent E. H.; Zhang S. Single-Step-Fabricated Disordered Metasurfaces for Enhanced Light Extraction from LEDs. Light: Sci. Appl. 2021, 10 (1), 180 10.1038/s41377-021-00621-7. 34489399 PMC8421350

[ref28] Koo W. H.; Jeong S. M.; Araoka F.; Ishikawa K.; Nishimura S.; Toyooka T.; Takezoe H. Light Extraction from Organic Light-Emitting Diodes Enhanced by Spontaneously Formed Buckles. Nat. Photonics 2010, 4 (4), 222–226. 10.1038/nphoton.2010.7.

[ref29] Do Y. R.; Kim Y. C.; Song Y. W.; Cho C. O.; Jeon H.; Lee Y. J.; Kim S. H.; Lee Y. H. Enhanced Light Extraction from Organic Light-Emitting Diodes with 2D SiO_2_/SiN_x_ Photonic Crystals. Adv. Mater. 2003, 15 (14), 1214–1218. 10.1002/adma.200304857.

[ref30] Qu Y.; Slootsky M.; Forrest S. R. Enhanced Light Extraction from Organic Light-Emitting Devices Using a Sub-Anode Grid. Nat. Photonics 2015, 9 (11), 758–763. 10.1038/nphoton.2015.194.

[ref31] Wrzesniewski E.; Eom S. H.; Cao W.; Hammond W. T.; Lee S.; Douglas E. P.; Xue J. Enhancing Light Extraction in Top-Emitting Organic Light-Emitting Devices Using Molded Transparent Polymer Microlens Arrays. Small 2012, 8 (17), 2647–2651. 10.1002/smll.201102662. 22678825

[ref32] Kim J. K.; Chhajed S.; Schubert M. F.; Schubert E. F.; Fischer A. J.; Crawford M. H.; Cho J.; Kim H.; Sone C. Light-Extraction Enhancement of GaInN Light-Emitting Diodes by Graded-Refractive-Index Indium Tin Oxide Anti-Reflection Contact. Adv. Mater. 2008, 20 (4), 801–804. 10.1002/adma.200701015.

[ref33] Xu Z.; Portnoi M.; Papakonstantinou I. Micro-Cone Arrays Enhance Outcoupling Efficiency in Horticulture Luminescent Solar Concentrators. Opt. Lett. 2023, 48 (1), 183 10.1364/OL.478206. 36563401

[ref34] Slootsky M.; Forrest S. R. Enhancing Waveguided Light Extraction in Organic LEDs Using an Ultra-Low-Index Grid. Opt. Lett. 2010, 35 (7), 1052 10.1364/OL.35.001052. 20364214

[ref35] Wierer J. J.; David A.; Megens M. M. III-Nitride Photonic-Crystal Light-Emitting Diodes with High Extraction Efficiency. Nat. Photonics 2009, 3 (3), 163–169. 10.1038/nphoton.2009.21.

[ref36] Agata K.; Murai S.; Tanaka K. Stick-and-Play Metasurfaces for Directional Light Outcoupling. Appl. Phys. Lett. 2021, 118 (2), 021110 10.1063/5.0034115.

[ref37] Kumar P.; Khanna A.; Son S. Y.; Lee J. S.; Singh R. K. Analysis of Light Out-Coupling from Microlens Array. Opt. Commun. 2011, 284 (19), 4279–4282. 10.1016/j.optcom.2011.06.005.

[ref38] Kim J.; Qu Y.; Coburn C.; Forrest S. R. Efficient Outcoupling of Organic Light-Emitting Devices Using a Light-Scattering Dielectric Layer. ACS Photonics 2018, 5 (8), 3315–3321. 10.1021/acsphotonics.8b00539.

[ref39] Möller S.; Forrest S. R. Improved Light Out-Coupling in Organic Light Emitting Diodes Employing Ordered Microlens Arrays. J. Appl. Phys. 2002, 91 (5), 3324–3327. 10.1063/1.1435422.

[ref40] Velten T.; Bauerfeld F.; Schuck H.; Scherbaum S.; Landesberger C.; Bock K. Roll-to-Roll Hot Embossing of Microstructures. Microsyst. Technol. 2011, 17, 619–627. 10.1007/s00542-010-1158-x.

[ref41] Ginting R. T.; Jeon E. B.; Kim J. M.; Jin W. Y.; Kang J. W. Dual Light Trapping and Water-Repellent Effects of a Flexible-Based Inverse Micro-Cone Array for Organic and Perovskite Solar Cells. ACS Appl. Mater. Interfaces 2018, 10 (37), 31291–31299. 10.1021/acsami.8b08669. 30133246

[ref42] Dottermusch S.; Schmager R.; Klampaftis E.; Paetel S.; Kiowski O.; Ding K.; Richards B. S.; Paetzold U. W. Micro-Cone Textures for Improved Light in-Coupling and Retroreflection-Inspired Light Trapping at the Front Surface of Solar Modules. Prog. Photovolt.: Res. Appl. 2019, 27 (7), 593–602. 10.1002/pip.3133.

[ref43] Kim J.; Battaglia C.; Charrière M.; Hong A.; Jung W.; Park H.; Ballif C.; Sadana D. 9.4% Efficient Amorphous Silicon Solar Cell on High Aspect-Ratio Glass Microcones. Adv. Mater. 2014, 26 (24), 4082–4086. 10.1002/adma.201400186. 24648188

[ref44] Wang D.; Sun Q.; Hokkanen M. J.; Zhang C.; Lin F. Y.; Liu Q.; Zhu S. P.; Zhou T.; Chang Q.; He B.; Zhou Q.; Chen L.; Wang Z.; Ras R. H. A.; Deng X. Design of Robust Superhydrophobic Surfaces. Nature 2020, 582 (7810), 55–59. 10.1038/s41586-020-2331-8. 32494077

[ref45] Jabeen F.; Chen M.; Rasulev B.; Ossowski M.; Boudjouk P. Refractive Indices of Diverse Data Set of Polymers: A Computational QSPR Based Study. Comput. Mater. Sci. 2017, 137, 215–224. 10.1016/j.commatsci.2017.05.022.

