# Supplementary material for: Optimizing Horticulture Luminescent Solar Concentrators via Enhanced Diffuse Emission Enabled by Micro-Cone Arrays
Source: ACS Appl Mater Interfaces. 2024 May 15;16(21):27587–95. doi: 10.1021/acsami.4c01707 (PMC11145591; doi:10.1021/acsami.4c01707)
Supplement: Supplementary file 1 — am4c01707_si_001.pdf [file am4c01707_si_001.pdf]

SUPPORTING INFORMATION

Optimizing Horticulture Luminescent Solar Concentrators via Enhanced Diffuse Emission Enabled by Micro-Cone Arrays

Zhijie Xu[1], Martyna Michalska[2], Ioannis Papakonstantinou[1]*

1 Photonic Innovations Lab, Department of Electronic and Electrical Engineering, University College London, London WC1E 7JE, UK.
2 Manufacturing Futures Lab, Department of Mechanical Engineering, University College London, Queen Elizabeth Olympic Park, London, E20 3BS, UK.

KEYWORDS: luminescent solar concentrators, horticulture, outcoupling efficiency, micro-cones, spectral conversion, bidirectional transmittance distribution function

*Corresponding author: i.papakonstantinou@ucl.ac.uk.

Ray path in micro-cone structure

To analyze the mechanism of light extraction improvement using the micro-cone structure, a model is built in LightTools, as shown in **Figure S1(a)**. The tip angle α is a parameter defined by the H/R ratio. For high H/R ratio cases, as shown in **Figure S1(b-c)**, if the incident angle β_1 is greater than the critical angle, rays would be reflected and hit the other side of the conical surface. The second incident angle β_2 would decrease, and so on. After several reflections, the incident angle would finally become smaller than the critical angle, allowing the ray to escape from the cone. For low H/R ratio cases, as shown in **Figure S1(d-e)**, if the incident angle is either too small or too large, rays would be reflected backward.

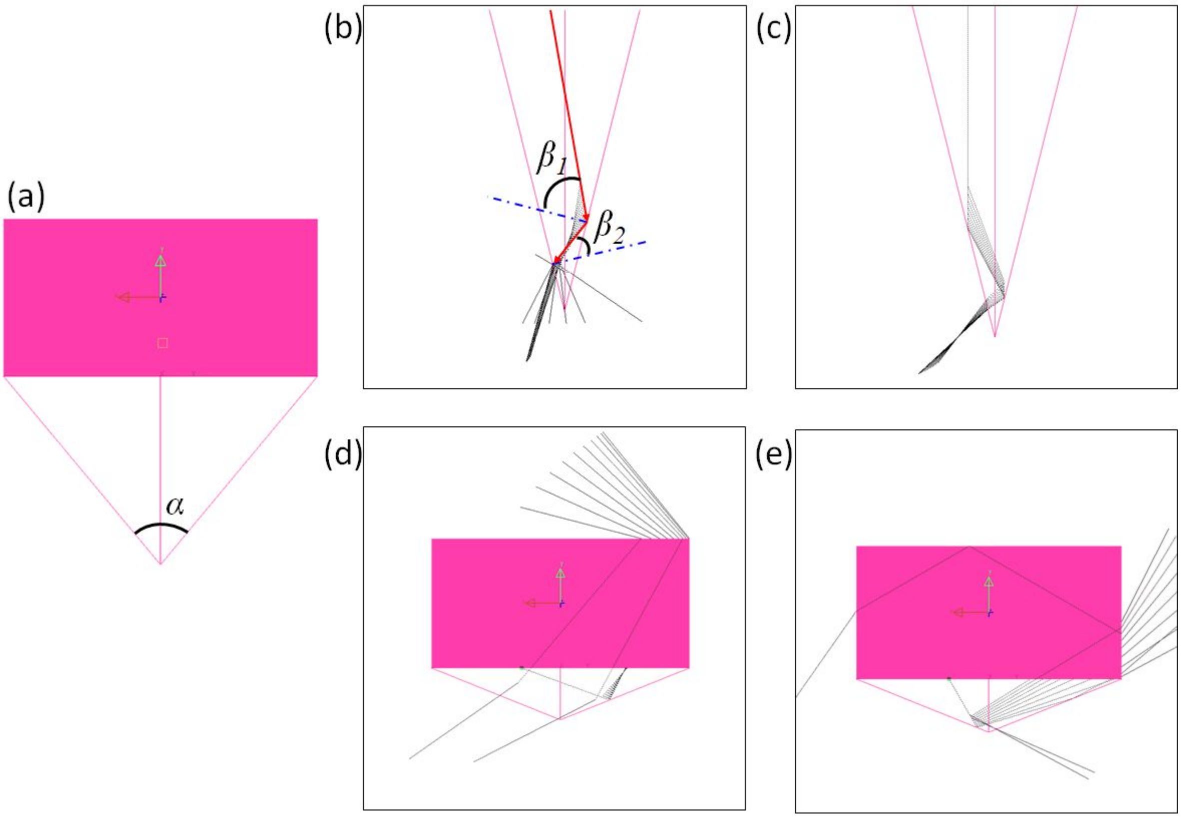

Figure S1. Analysis of ray path based on Monte Carlo ray tracing. a) Micro-cone structure model. b-c) Ray paths for high H/R ratio micro-cone structure. Blue dash-dot lines represent the normal vector of the conical surface. d-e) Ray paths for low H/R ratio micro-cone structure.

Internal and external photon fates

Monte Carlo ray tracing method was employed to calculate the internal and external photon fates, as depicted in **Figure S2**. The internal quantum efficiency (IQE) exhibits an increase as the H/R ratio increases, eventually saturating at approximately 40% for H/R>1. In addition, a maximum in absorbance is observed when H/R=1.2. The combination of the above two conditions results in the external quantum efficiency (EQE) peaking for H/R=1.2, as we mentioned in our paper.

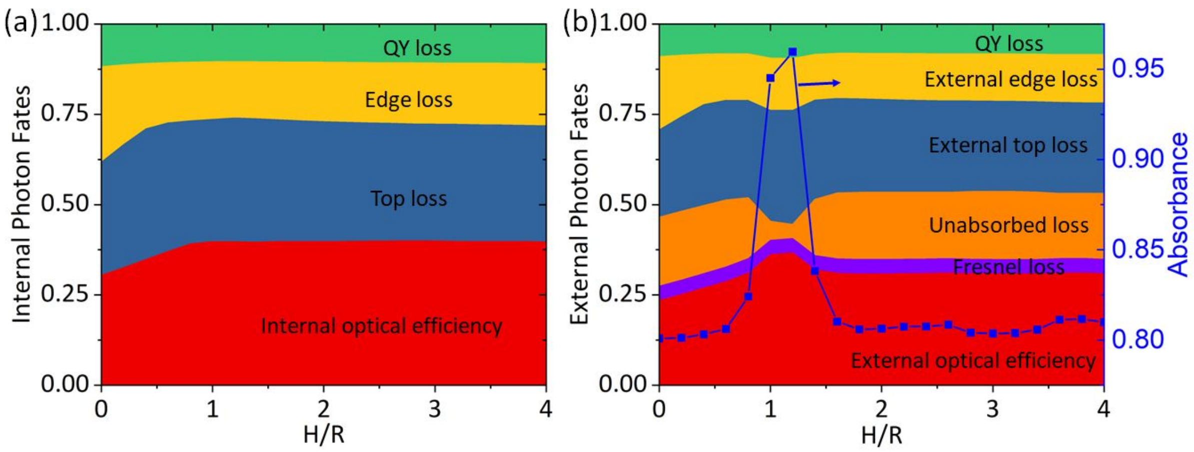

Figure S2. a) Internal and b) external photon fates based on different H/R ratios. Blue line in b illustrates the absorbance variation.

Micro-cone array morphologies

Micro-cone array morphologies at different stages are illustrated in **Figure S3**. As an illustration, we utilized the sample with an H/R ratio of 0.4 to demonstrate the alterations in morphology. The nanoprinted master on the Si wafer is depicted in **Figure S3(a)**. The imprinted protruding outcome is showcased in **Figure S3(b)**, showcasing an inverted cone profile relative to the master. Similarly, the result obtained through nanoimprint lithography (NIL) based on the master is exhibited in **Figure S3(c)**. Finally, the imprinted extruding micro-cone arrays, derived from the NIL result, are presented in **Figure S3(d)**.

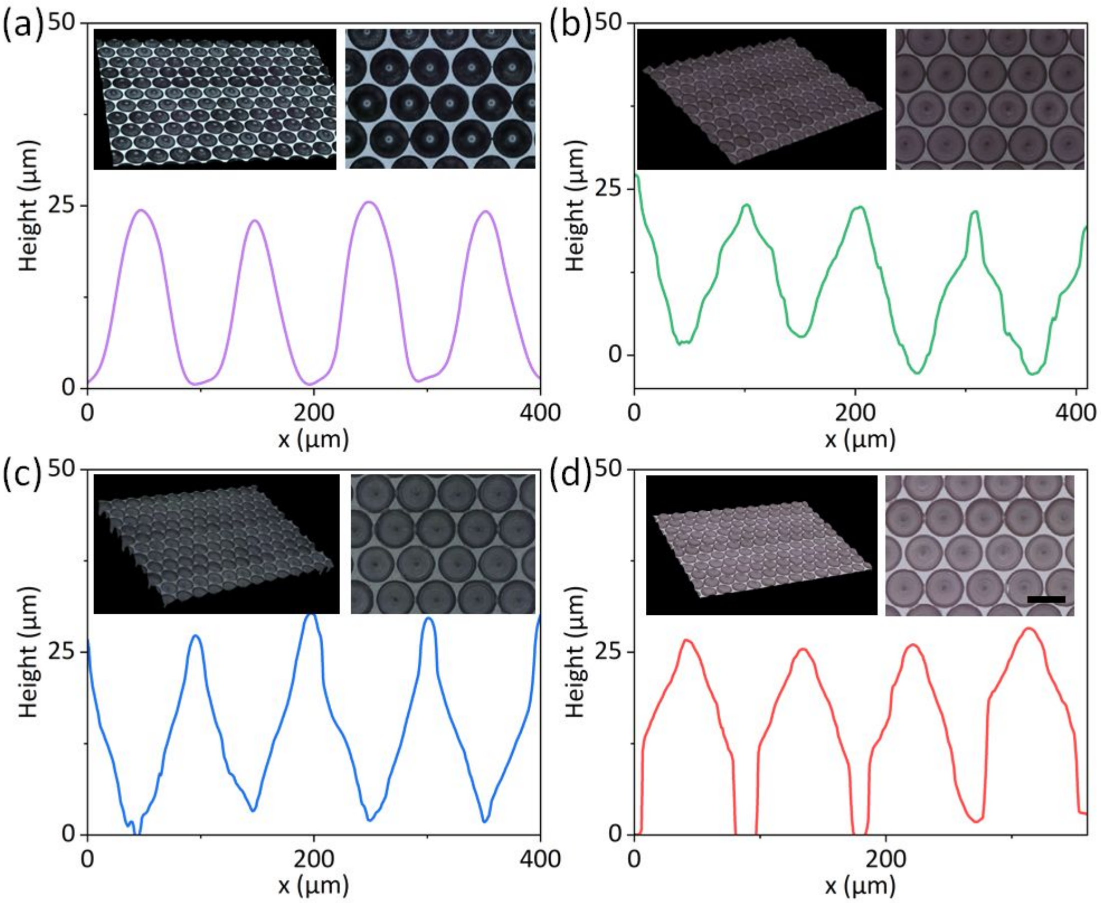

Figure S3. Micro-cone array morphologies. a) Micro-cone arrays patterned using nanoprinting. b) Protruding micro-cone arrays obtained through imprinting from a. c) Micro-cone arrays produced through nanoimprint lithography based on a. d) Extruding micro-cone arrays resulting from imprinting of c. All inset figures share a common scale bar of 100 μm.

Internal photon fates under different incident angles

Compared with external efficiency, internal efficiency better depicts the performance of light extraction. Hence, we use a Monte Carlo ray tracing method to evaluate the internal photon fates of HLSC under different incident angles, as shown in **Figure S4**. Apparently, all the photon fates, including QY loss, edge loss, top loss, and IQE, remain almost unchanged when the incident angle varies from normal (0°) to 80°.

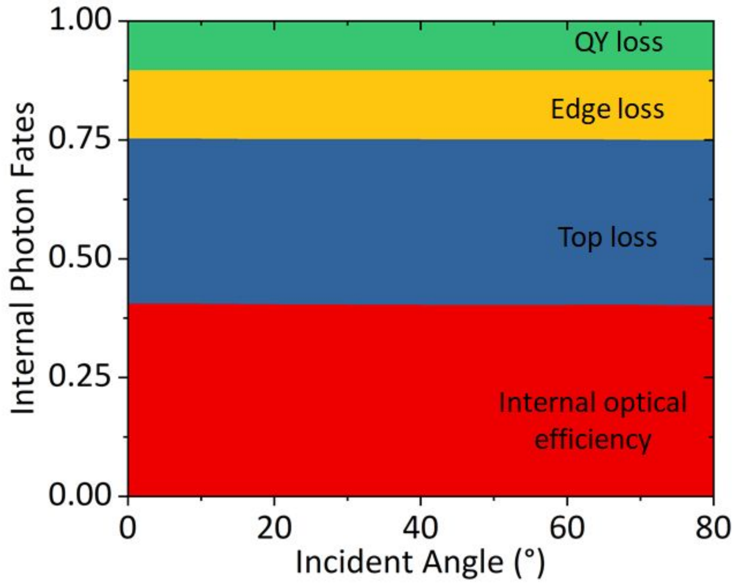

Figure S4. Internal photon fates under different incident angles.

Concentrations of HLSC

In our investigations, we employed a Monte Carlo ray tracing method to assess the behavior of HLSC across varying concentrations. To facilitate comparisons, we designated a concentration of 1×10^{-4} M as the unit concentration. Thereafter, we employed a scale factor to illustrate concentration increments (e.g., a scale factor of 2 corresponds to 2×10^{-4} M). The findings are presented in **Figure S5**.

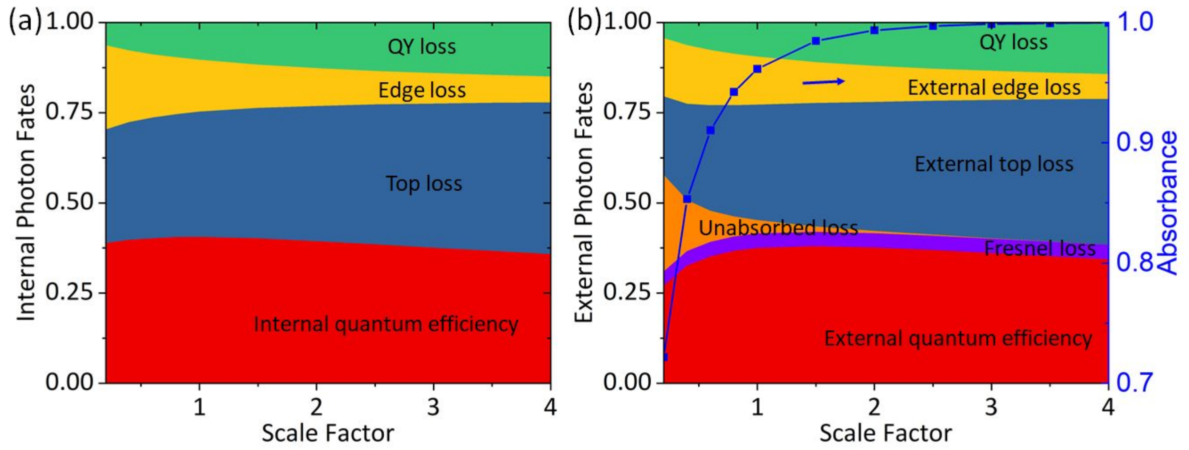

Figure S5. a) Internal and b) external photon fates based on different concentrations. Blue line in b illustrates the absorbance variation.

Radiation angle and insolation calculation

As illustrated in **Figure S6**, various characteristic angles are associated with solar radiation. For the sake of simplicity, we assume that the device is oriented perpendicularly to the south, optimizing its exposure to solar radiation. In this context, angle γ in **Figure S1** is equivalent to 0°. The roof slope is represented by angle β in **Figure S1**. We define the angle θ as the radiation angle, which denotes the angle between the normal to the roof and the incident beam. The radiation angle can be calculated using the following formula[1]:

$\cos\theta = \cos\theta_z \cos\beta + \sin\theta_z \sin\beta \cos\gamma_s$, (s1)

where $\theta_z = 90° - \alpha_s$. α_s is the solar altitude angle. γ_s is the azimuth angle. γ_s could be calculated by[1]:

$\sin\gamma_s = \frac{\cos\delta \sin\omega}{\cos\alpha_s}$, (s2)

where δ is the solar declination and ω is hour angle. These parameters could be calculated by[1]:

$\delta = 23.45 \cdot \sin\frac{360(284+n)}{365}$, (s3)

where n is the day of year. Solar altitude angle is given by[1]:

$cos\theta_s = sin\,\alpha_s = sin\,\phi sin\,\delta + cos\,\phi cos\,\delta cos\,\omega$, (s4)

where ϕ is the latitude.

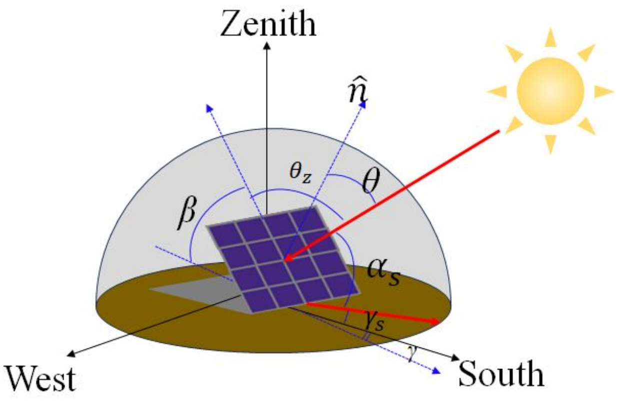

Figure S6. Characteristic angles for solar radiation. $\hat{n}$ represents the normal vector of the roof surface.

For solar irradiation, our primary focus is on clear-sky direct beam radiation, which is expressed by the following equation[2]:

$i(h) = a \cdot e^{-k \cdot r(h)}$, (s5)

where a is the apparent solar irradiation on day n[2]:

$a = 1160 + 75\sin(\frac{2\pi}{365}(n - 275))$. (s6)

And k is the optical depth[2]:

$k = 0.174 + 0.035\sin(\frac{2\pi}{365}(n - 100))$. (s7)

And r is the air mass raio[2]:

$r(h) = \sqrt{[708\sin(\alpha_s(h))]^2 + 1417} - 708\sin(\alpha_s(h))$. (s8)

Insolation variation

The variation in insolation throughout the year in London was calculated. Considering that roof slopes typically range from 30° to 60°, the insolation variation for angles of 30°, 40°, 50°, and 60° is illustrated in **Figure S7**. The X-axis in all figures represents the hours of the day from 1:00 to 24:00, and the Y-axis represents the days of the year from January 1st to December

31st. The solid black lines inset serve as contour lines for a 45° incident angle. It's evident that if the incident angle falls above 45°, there is a significant reduction in insolation.

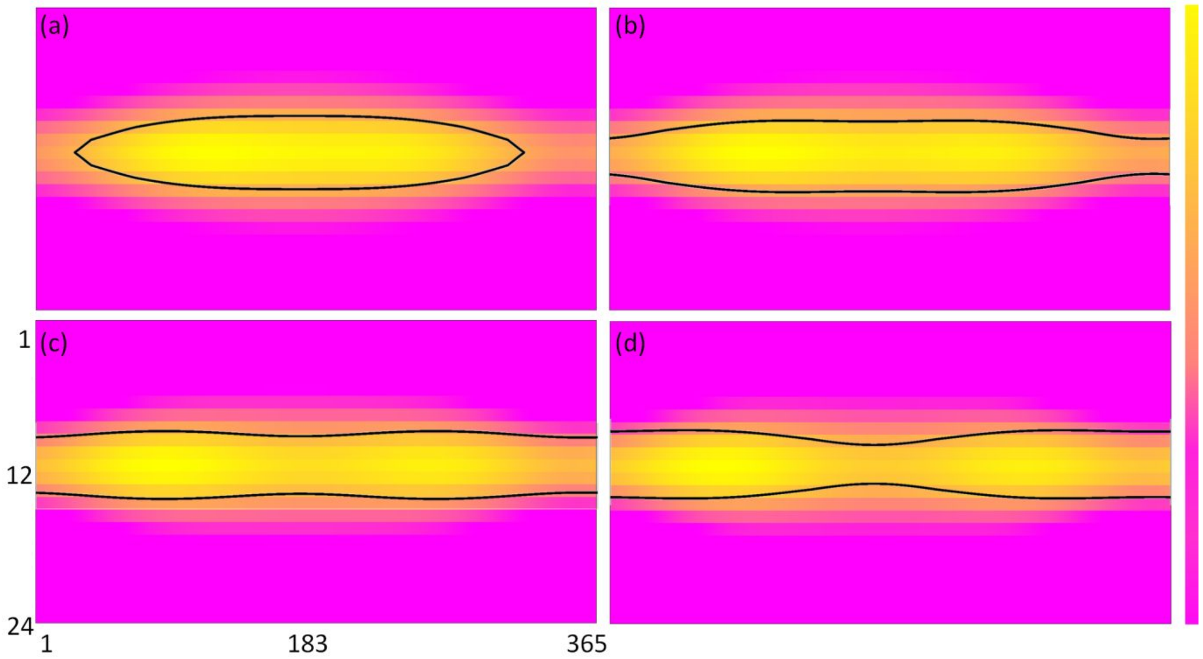

Figure S7. Insolation variation throughout the year in London calculated under different roof slopes: a) 30°; b) 40°; c) 50° and d) 60°.

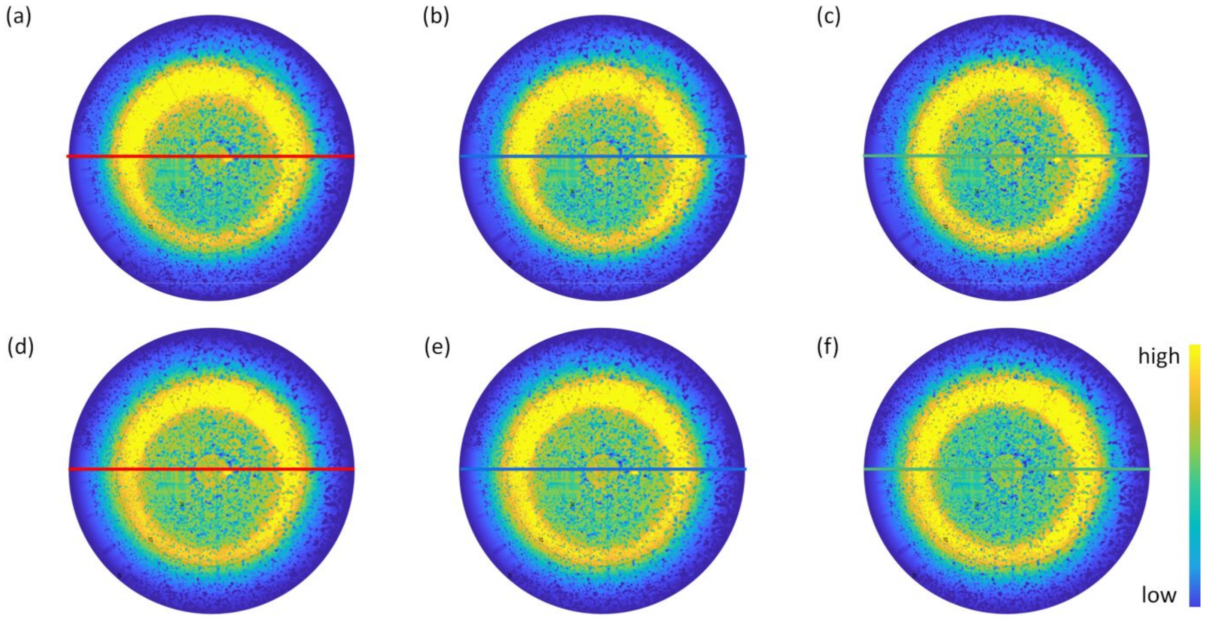

Figure S8. Measured BTDF in polar coordinates for a-c) extruding and d-f) protruding micro-cone arrays. a and d are measurded under an incident angle of 10°. b and e are measurded under an incident angle of 20°. c and f are measurded under an incident angle of 30°.

Monte Carlo ray tracing

Monte Carlo ray tracing is a widely used method in LSC research[3–11]. We employed a commercial software called LightTools (Optical Research Associates) to analyze the performance of HLSC. This method allows for the collection of both outcoupling efficiency and irradiance distributions. The HLSC model is constructed based on an untextured LSC, as depicted in **Figure S9**. A thin film with dimensions of 60×60×5 mm^3 serves as the host matrix. Its refractive index is set at 1.5, which is a common index for polymer materials[12]. The properties of the fluorophores within the matrix are defined using parameters such as mean free path (MFP), quantum yield (QY), absorption spectrum, and emission spectrum. These data are obtained experimentally through the characterization of an HLSC doped with Lumogen Red.

Micro-cone arrays are patterned by adding texture to the bottom surface of the planar LSC, as shown in **Figure S9**. Extrusion is achieved by defining the texture as a bump, while protrusion is defined as a hole. The micro-cone arrays cover the entire bottom surface. The light source is defined as a parallel source, and its direction can be continuously adjusted for analysis.

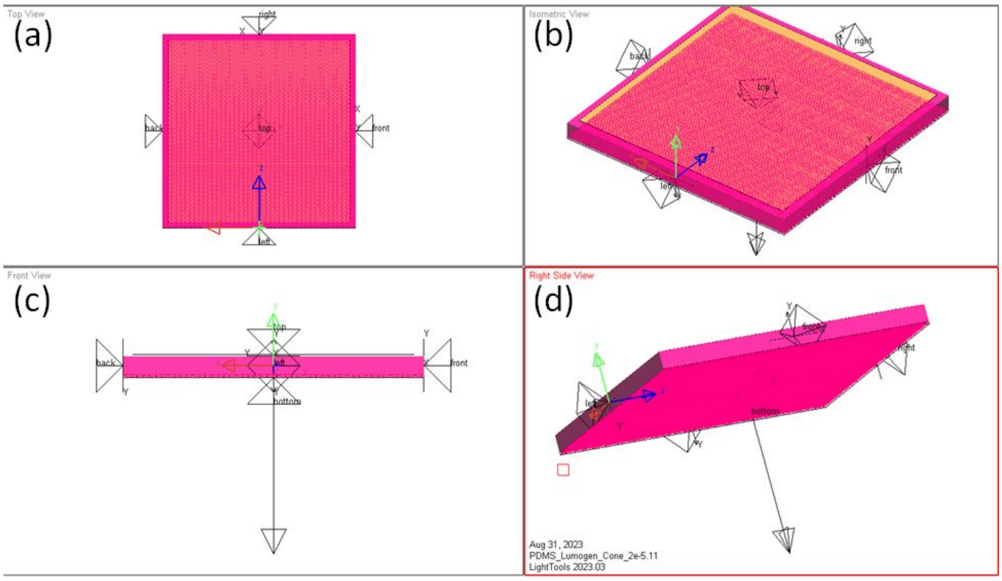

Figure S9. HLSC modelling using LightTools. a) Top view. b) Isometric view. c) Front view. d) Right side view.

Simulated HLSC BTDF

HLSC BTDF are simulated using the Monte Carlo ray tracing method. Simulated results for different H/R ratios are illustrated in **Figure S10**. All these results are normalized to the highest irradiance for comparison. It is noteworthy that these simulated irradiances closely match the experimental results, indicating the reliability and accuracy of the simulation approach.

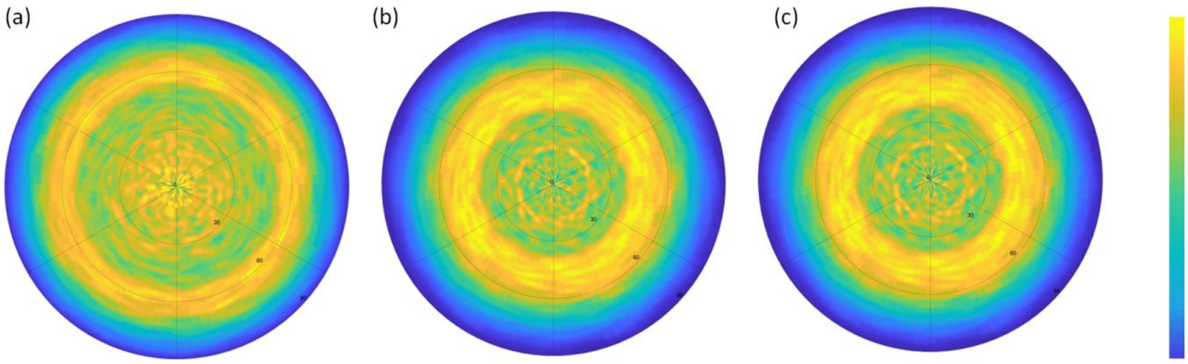

Figure S10. Simulated HLSC BTDF for different H/R ratios a) 0.4; b) 1.2 and c) 2.0.

Light stability

To validate the light stability of our sample, it was subjected to a UV resistance test within a chamber. The sample was continuously exposed to two UV lamps, as depicted in Figure S11 a. The local power of the UV light was measured to be 7.5 mW using a power sensor (Thorlabs, S405C). Considering the diameter of the sensor is 1 cm, the intensity of the radiation corresponds to 95.5 W/m^2. The variation in QY was recorded over a period of 15 days, equivalent to solar radiation exposure near the equator for about 4.5 months. The results indicate that the QY remained almost unchanged during the UV resistance test, providing evidence of the excellent light stability of our sample.

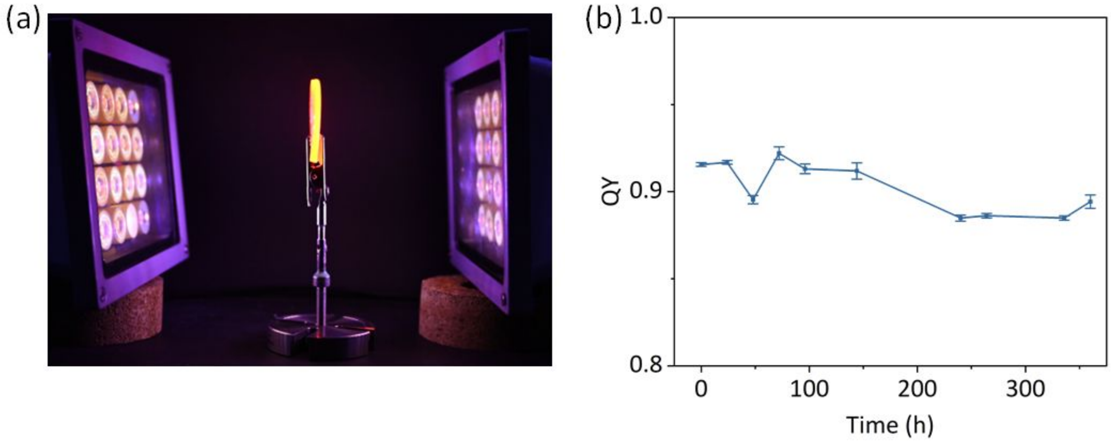

Figure S11. Light stability. a) Setup of UV resistance test. b) QY variation over a 15-day period.

Reference

(1) Kalogirou, S. A. 3.01 - Solar Thermal Systems: Components and Applications—Introduction. In *Comprehensive Renewable Energy*; Elsevier, 2022; Vol. 1–9, pp 1–25.

(2) Masters, G. M. *Renewable and Efficient Electric Power Systems*; 2013.

(3) Papakonstantinou, I.; Tummeltshammer, C. Fundamental Limits of Concentration in Luminescent Solar Concentrators Revised: The Effect of Reabsorption and Nonunity Quantum Yield. *Optica* **2015**, *2* (10), 841.

(4) Xu, Z.; Portnoi, M.; Papakonstantinou, I. Micro-Cone Arrays Enhance Outcoupling Efficiency in Horticulture Luminescent Solar Concentrators. *Opt Lett* **2022**, *48* (1), 183–186.

(5) Portnoi, M.; Sol, C.; Tummeltshammer, C.; Papakonstantinou, I. Impact of Curvature on the Optimal Configuration of Flexible Luminescent Solar Concentrators. *Opt Lett* **2017**, *42* (14), 2695.

(6) Tummeltshammer, C.; Taylor, A.; Kenyon, A. J.; Papakonstantinou, I. Flexible and Fluorophore-Doped Luminescent Solar Concentrators Based on Polydimethylsiloxane. *Opt Lett* **2016**, *41* (4), 713.
(7) Tummeltshammer, C.; Taylor, A.; Kenyon, A. J.; Papakonstantinou, I. Homeotropic Alignment and Förster Resonance Energy Transfer: The Way to a Brighter Luminescent Solar Concentrator. *J Appl Phys* **2014**, *116* (17).
(8) Tummeltshammer, C.; Brown, M. S.; Taylor, A.; Kenyon, A. J.; Papakonstantinou, I. Efficiency and Loss Mechanisms of Plasmonic Luminescent Solar Concentrators. *Opt Express* **2013**, *21* (S5), A735.
(9) Portnoi, M.; Haigh, P. A.; Macdonald, T. J.; Ambroz, F.; Parkin, I. P.; Darwazeh, I.; Papakonstantinou, I. Bandwidth Limits of Luminescent Solar Concentrators as Detectors in Free-Space Optical Communication Systems. *Light Sci Appl* **2020**, *10* (1), 1–12.
(10) Portnoi, M.; Macdonald, T. J.; Sol, C.; Robbins, T. S.; Li, T.; Schläfer, J.; Guldin, S.; Parkin, I. P.; Papakonstantinou, I. All-Silicone-Based Distributed Bragg Reflectors for Efficient Flexible Luminescent Solar Concentrators. *Nano Energy* **2020**, *70* :104507.
(11) Tummeltshammer, C.; Portnoi, M.; Mitchell, S. A.; Lee, A. T.; Kenyon, A. J.; Tabor, A. B.; Papakonstantinou, I. On the Ability of Förster Resonance Energy Transfer to Enhance Luminescent Solar Concentrator Efficiency. *Nano Energy* **2017**, *32*, 263–270.
(12) Jabeen, F.; Chen, M.; Rasulev, B.; Ossowski, M.; Boudjouk, P. Refractive Indices of Diverse Data Set of Polymers: A Computational QSPR Based Study. *Comput Mater Sci* **2017**, *137*, 215–224.
